# Investigation of ethics approval as part of a research integrity assessment of randomised controlled trials in COVID-19 evidence syntheses: a meta-epidemiological study

**DOI:** 10.1136/bmjopen-2024-092244

**Published:** 2025-03-24

**Authors:** Florencia Weber, Tamara Pscheidl, Emma Sydenham, Patrick Meybohm, Stephanie Weibel

**Affiliations:** 1 Intensive Care, Emergency and Pain Medicine, Department of Anaesthesiology, University Hospital Würzburg, Würzburg, Germany; 2 Cochrane Central Editorial Service, Cochrane, London, UK

**Keywords:** Systematic Review, MEDICAL ETHICS, STATISTICS & RESEARCH METHODS

## Abstract

**Abstract:**

**Objectives:**

Ethical compliance of randomised controlled trials (RCTs) documented as ethics committee (EC) approval is vital for participant protection but is often overlooked by evidence synthesis producers despite regulatory mandates. We aimed to systematically assess reporting of ethics approval and informed consent (IC) in RCTs included in evidence syntheses and examined its potential impact on the study pool as part of a research integrity assessment (RIA).

**Design:**

Meta-epidemiological study.

**Setting:**

Assessment of ethics approval; domain 3 of the RIA tool developed for evidence syntheses.

**Participants/subjects:**

COVID-19 RCTs included in evidence syntheses.

**Primary outcomes:**

We extracted ethical items from study reports, that is, ethics approval statements, EC details, ethics approval numbers (ENs), IC and verified national recognition of ECs. RCTs were assessed regarding ethics approval and categorised as ‘no concern’, ‘awaiting classification’ or ‘exclude’ from the study pool. We also examined the impact of study settings on ethics approval reporting and discussed assessment reliability.

**Results:**

We included 188 RCTs. 93% of primary study reports contained an ethics statement, 70% provided EC details, 44% reported EN and 91% mentioned IC. Trial registration records identified the EC in 8 RCTs and EN in 25 RCTs. Overall, 41% of RCTs reported all ethical items. Authors of 95 RCTs were contacted for missing information, yielding 22 satisfactory responses. Of the 151 RCTs with identified ECs, 88% were nationally recognised. Overall, 53% of RCTs were classified as ‘no concern’, 47% as ‘awaiting classification’ and none were excluded. Most were ‘awaiting classification’ due to reporting-related reasons. No significant differences in ethics approval reporting were observed across study settings, countries, or sample sizes.

**Conclusions:**

Reporting of ethical items in RCTs remains inadequate. Including ethics approval details in reporting guidelines such as Consolidated Standards of Reporting Trials could improve this. Current under-reporting issues limit the reliability of the RIA tool’s ethics approval assessment.

**Protocol registration:**

The protocol is available on OSF (https://osf.io/3bzeg).

**STRENGTHS AND LIMITATIONS OF THIS STUDY**

- The study uses a meta-epidemiological approach to systematically assess ethics approval in COVID-19 randomised controlled trials using both primary study reports and trial registration records for a comprehensive evaluation.
- Active engagement with study authors to obtain missing information or clarify inconsistencies improves the accuracy and completeness of the ethics approval assessments.
- The reliance on reported data in study publications and trial registrations, which may be incomplete or inaccurate, limits the reliability of the ethics approval assessment.
- The absence of standardised, internationally recognised procedures for reporting ethics approval and other ethical items complicates consistent and accurate assessment across diverse studies.
- The lack of a global registry for ethics committees hinders the verification of their national recognition status, and therefore the assessment of ethics approval of studies.

## Background

 The basis for reliable results in evidence syntheses is the knowledge of the trustworthiness of the underlying research evidence base. Research that follows the principles of research integrity (RI) ensures trustworthiness. To date, producers of evidence syntheses have not routinely assessed the RI of studies included in their evidence syntheses. Critical appraisal tools, such as Cochrane Risk of Bias tool 2 and Grading of Recommendations, Assessment, Development and Evaluation, used to assess the internal and external validity of study results, do not necessarily address aspects of RI.^1 2^ There is an ongoing debate on how to appraise RI, and several projects are ongoing to develop trustworthiness screening and research integrity assessment (RIA) tools for producers of evidence syntheses.^3^,^6^

Most researchers associate RI with the use of honest and verifiable methods in proposing, performing and evaluating research, but RI also comprises adhering to (inter)national and commonly accepted guidelines, regulations, norms and standards.^7^ Clinical studies should follow good clinical practice (GCP)—a code of international ethical and scientific standards for designing, recording and reporting studies that involve the participation of human subjects.^8^ The World Medical Association has developed the Declaration of Helsinki, which states that “physicians must consider the ethical, legal and regulatory norms and standards for research involving human subjects in their own countries as well as applicable international norms and standards” […] and that a “research protocol must be submitted for consideration, comment, guidance and approval to the concerned [independent] research ethics committee before the study begins”.^9^ We distinguish between the ‘ethical compliance’ of a clinical trial, which refers to adherence to research ethics as determined by ethics committees (ECs), and the process of ‘obtaining ethical approval’ by study investigators or funders.^10^

Compliance of clinical studies with ethics approval standards can only be reliably assessed if details are fully reported in study reports. Unfortunately, the reporting of ethics approval in randomised controlled trials (RCTs) is currently not included as an item in the Consolidated Standards of Reporting Trials (CONSORT 2010) statement.^11^ Given the lack of standardisation in reporting, it is unclear how ethics approval of RCTs should be assessed within an evidence synthesis and what impact an assessment may have on the results and conclusions of evidence syntheses.

This article is part of a meta-epidemiological study which applies a novel and non-validated tool, designed for a RIA of interventional RCTs of an investigational medicinal product (IMP) in the context of an evidence synthesis,^12^ to a pool of RCTs included in COVID-19 systematic reviews. In this part, we focus on the assessment of the third domain of the RIA tool, that is, ethics approval of RCTs. We present reporting of ethics approval and IC in the study reports of recent RCTs, provide guidance for producers of evidence synthesis on how to assess ethics approval and discuss the feasibility of the tool for its use in evidence synthesis.

## Methods

The protocol for the meta-epidemiological study has been published, including the search for RCTs and the assessment of ethics approval (https://osf.io/3bzeg). We extracted and analysed additional study data, which was not prospectively planned, but designed post hoc to describe the study pool in detail. Additional analyses are indicated as such.

### Selection of RCTs for assessment with the RIA tool

We searched for Cochrane reviews (CRs) and non-Cochrane systematic reviews (SRs) with or without meta-analysis evaluating 13 interventions for the prevention or treatment of SARS-CoV-2 infection and COVID-19 in humans, irrespective of SARS-CoV-2 diagnosis, disease severity or treatment setting. Pairwise and network meta-analyses were eligible. We included full-text, peer-reviewed journal publications of systematic reviews. Preprints of systematic reviews, scoping reviews and narrative reviews were not eligible. We restricted the inclusion to publications in English. Further details on the inclusion criteria of CRs and SRs in terms of population, interventions and comparators are described in the protocol (https://osf.io/3bzeg).

Two reviewers independently searched for all eligible CRs and SRs with regard to study design, population and relevant interventions in PubMed to 9 June 2022. The search strategy is provided in the protocol (https://osf.io/3bzeg). One reviewer selected the CR (or its update) and the SR (or its update) to each of the relevant interventions with the largest RCT pool based on the most recent search date or the broadest inclusion criteria. The study pool of RCTs for further testing of RIA consisted of the primary studies included in the eligible systematic reviews. RCTs published as journal publications, preprints or unpublished with results posted in trial registries were eligible. Depending on the type of published results, either journal publications, preprints or trial registration records were considered ‘primary study reports’. Multiple primary study reports of a study (eg, journal publication and preprint) were not pooled for our assessment but were separately assessed as included in the original systematic review.

In the present study, we excluded retracted RCTs (ie, first domain of the RIA) and studies which were incorrectly included in the selected evidence syntheses as RCTs, although the studies clearly stated that a non-randomised study design was used. All RCTs, which were not previously excluded, were assessed in this study, irrespective of the registration status (ie, second domain of the RIA). We documented the screening and selection process of systematic reviews and RCTs in a Preferred Reporting Items for Systematic Reviews and Meta-Analyses flow diagram, including reasons for exclusion at the full-text screening stage.

### Data extraction of study characteristics

One reviewer (ie, the third reviewer in the meta-epidemiological RIA study, FW) extracted details on ethics approval for all RCTs included in this study from the primary study reports, supplemental materials, study protocols and trial registration records to September 2023. Where available, original data extractions made by two independent reviewers on ethics approval in the RIA study were used and checked by the third reviewer (FW). If double extracted data were not available (ie, for RCTs which previously did not pass domains 1 and 2 of the RIA), or if discrepant extractions between pairs of reviewers occurred, a third reviewer (FW) extracted missing data or solved conflicts for this study.

Originally, the third domain of the RIA on ethics approval included five items for the assessment of RCTs,^12^ that is, reporting of an ethics approval statement in the primary study report, name and location of the EC (or alternatively named, ‘institutional review board’ (IRB), ‘research ethics committee’ (REC)), national recognition status of the EC according to country, ethics approval number (EN) and informed consent (IC). In this study, one reviewer (FW) additionally extracted the date of the ethics approval (ie, date of ethics opinion) and the date of study start as reported in the primary study report and in the trial registration records. We also extracted the following information from all RCTs, that is, sample size, setting (single-centre vs national multicentre vs international multicentre), location (ie, country) where the study was conducted and the trial registry with registration number where the study was registered.

### Assessment of ethics in RCTs

#### Statement on ethics approval

We investigated whether the RCTs included an informative statement on ethics approval in the primary study report. Every statement on ethics approval was counted if it at least mentioned that an EC granted the approval (eg, approved by an institutional EC) or the EN was reported. The EC did not have to be named. Generic declarations, such as “this study was conducted in accordance with the Declaration of Helsinki and/or with the local regulations”, were considered insufficient. If no (sufficient) statement on ethics approval was reported in the primary study document, we contacted the study authors via email for further information.

#### Name and location of ethics committee

We assessed whether RCTs reported the name and location of at least one EC in the primary study report. We accepted reporting of at least one EC as sufficient for single-centre as well as for multicentre RCTs. Generic statements, for example, “ethics approval obtained from several IRBs, according to site”, without further specifications were considered as insufficient. If the name and location of the EC were not reported in the primary study report, we searched for the corresponding trial registration record of registered RCTs, and if not successful, we contacted the study authors via email for further information.

#### Recognition status of ethics committees

Interventional RCTs of an IMP in humans need ethics approval (ie, ethics opinion) by an authorised institution such as an EC.^9 13^ The EC should have a national recognition status, and there should be a national government department responsible for recording the committee’s national recognition status. To determine the recognition status of a reported EC, a new strategy was developed and applied by the third reviewer (FW), which was not included in the original RIA. We conducted a primary search in Google (https://www.google.de) following a standardised procedure, where the search was initiated by introducing the name and address of the EC as reported, supplemented by keywords, such as ‘ethics committee’ or ‘EC’, ‘institutional review board’ or ‘IRB’, or ‘research ethics committee’ or ‘REC’. With this primary search (latest search on 27 July 2023), we could identify national registries or directories of ECs for a total of 22 countries described in our RCT pool, which were provided by official organisations (ie, the Ministry of Health or bioethics or research entities). These directories were considered reliable sources. Additionally, four international directories listing local and independent ECs/IRBs and ethics regulations of various countries were also retrieved: two country-specific online databases (for low- and middle-income countries: https://healthresearchwebafrica.org.za/en; and for Europe: http://www.eurecnet.org/index.html), which provide clinical research regulatory information (ie, up-to-date ethical regulations, list of accredited EC/IRB); one online database of the Association for the Accreditation of Human Research Protection Programs, Inc. (https://www.aahrpp.org/), which provides current information about their independently accredited entities worldwide; as well as the website of the WHO Research Ethics Review Committee (https://www.who.int/groups/research-ethics-review-committee). All national and international sources are listed in online supplemental additional file 1.

In cases where an EC/IRB could not be located in any of the sources on the list, indirect searches through the website of the institution where the study took place were performed. An independent EC/IRB was considered ‘recognised’ if the institution provided a statement and/or documents proving national accreditation. These ECs/IRBs were also added to the list of sources (online supplemental additional file 1). If the search still remained futile, the recognition status of the EC/IRB was deemed to be ‘unclear’.

#### Ethics approval number

We assessed whether RCTs reported at least one EN in the primary study report. We accepted reporting of at least one EN as sufficient for single-centre as well as for multicentre RCTs. If no EN was reported in the primary study report, we searched the trial registration record of registered RCTs, and, if not successful, we contacted the study authors via email for further information.

#### Informed consent

We assessed if the primary study report included a statement on whether the RCT has obtained written IC (including electronically signed IC forms) from all participants or their relatives prior to enrolment. We also accepted verbal IC, if explicitly approved by an EC. In case of an insufficient statement or the lack of it, we contacted the study authors via email for further information.

### RIA judgement of RCTs considering ethics

Studies that sufficiently reported and fulfilled all of the original five RIA items were assessed as ‘no concern’ (ie, considered eligible for evidence synthesis).^12^ Studies with at least one item assessed as insufficient (ie, unclear/not found/not reported) were classified as ‘awaiting classification’ (ie, considered ineligible for evidence synthesis until clarification).^12^ Since there is no standardised recommendation for the reporting of ethical considerations in an RCT such as the CONSORT 2010 statement,^11^ we decided not to exclude any RCT due to missing information. However, studies with confirmed lack of an ethics approval in general or lack of IC were assessed as ‘exclude’ (ie, considered ineligible for evidence synthesis).

Authors of the RCTs classified as ‘awaiting classification’ were contacted in order to obtain missing information and clarify inconsistencies or concerns. Authors of unpublished RCTs (ie, only trial registration records available) were not contacted since those studies cannot be adequately assessed with current RIA items comparing journal publications or preprints with trial registration records. Authors had 14 days to respond. If a study author provided complete information, the RCT was upgraded to ‘no concern’. Study authors who did not provide any feedback were reminded via email and were given an additional 7 days to reply. The categorisation of the RCTs remained ‘awaiting classification’, if incomplete or no response was received.

For presentation in this study, we differentiated two subcategories of reasons for ‘awaiting classification’ regarding ethics, that is, RI-related and reporting-related reasons. We considered an RI-related reason to be the non-existence of an EC (ie, EC reported, but not found in our sources or an inability to establish the existence of the EC from any source). Reporting-related reasons included lack of or insufficient statement on ethics approval or EC, non-reporting of EN and unclear or not reported IC. However, it should be noted that non-reporting itself is problematic in terms of RI.

### Date of ethics approval

In this study, we also extracted and assessed additional items related to ethics approval, which were not included in the original RIA, that is, the date of ethics approval (also defined as the date of ethics opinion) and study start, and the timing of ethics approval. Additionally extracted and assessed items did not change the RI assessment for the ethics approval domain in this study. We assessed whether RCTs reported the date of ethics approval in the primary study report. We accepted reporting of at least one ethics approval date as sufficient for single-centre as well as for multicentre RCTs. A generic statement, for example, “ethics approval has been obtained prior to enrollment of the first subject”, was considered insufficient if no further details pertaining to the date of the ethics approval were disclosed. If the ethics approval date was not reported in the primary study report, we searched for the corresponding trial registration record of registered RCTs. For all RCTs, where we identified an ethics approval date, we assessed whether the ethics approval was obtained before the start of the study (ie, prospectively). We used the dates referred to as ‘study start’ as defined by different trial registry platforms and the study start date reported in the publication or preprint. Several RCTs reported different dates of study start and ethics approval as they took place in several locations (ie, conducted as multicentre RCTs). An RCT was considered having obtained a prospective ethics approval if true for at least one site. An ethics approval date after study start was considered either as retrospective ethics approval in case of single-centre settings or as ‘potentially retrospective’ in case of multicentre settings.

### Statistical analysis and presentation of data

This study has been designed to facilitate a descriptive data analysis. We did not perform any statistical hypothesis testing as this part of the study was not prospectively planned, but designed post hoc to disseminate relevant findings. We compared the categories of RCTs assessed as ‘no concern’, ‘awaiting classification’ and ‘exclude’, regarding reporting of ethics approval date, setting, location, sample size and trial registry. Descriptive statistics and frequency tables were used to present and compare variables (eg, sample size, setting, location, and trial registry).

Due to the large number of studies, we only referenced individual studies in the following results section if less than 10 studies are referred to. We restricted referencing of studies to our investigations on the original ethics items for RIA. Data and digital object identifiers (doi) for all individual studies are available online (data set).^14^

Due to the large number of studies, we only referenced individual studies in the following results section if less than 10 studies are referred to. We restricted referencing of studies to our investigations on the original ethics items for RIA. Data and digital object identifiers (doi) for all individual studies are available online (data set).^14^

Due to the large number of studies, we only referenced individual studies in the following results section if less than 10 studies are referred to. We restricted referencing of studies to our investigations on the original ethics items for RIA. Data and digital object identifiers (doi) for all individual studies are available online (data set).^14^

### Patient and public involvement

Patients and/or the public were not involved in the design, or conduct, or reporting, or dissemination plans of this research.

## Results

A total of 206 RCTs included in 23 evidence syntheses (ie, 13 CRs and 10 SRs, online supplemental additional file 2) investigating interventions of interest for the treatment or prevention of SARS-CoV-2 infection were identified by our search (online supplemental additional file 3). We included 188 RCTs in this study and excluded 8 retracted RCTs and 10 studies which turned out to be non-randomised studies. Of 188 RCTs, 149 were published in journals, 33 were published on a preprint server and the remaining 6 RCTs were unpublished with results only posted on a trial registration database. References and all baseline details of included RCTs reported in the following (ie, ethical items, sample size, setting, country and trial registration) are available online (data set).^14^

Of 188 RCTs, 174 published and 1 unpublished RCT included an informative statement on ethics approval in the primary study report (table 1). Three published RCTs have not reported any statement on ethics approval (Kishoria-2020, Li-2021, Mareev-2021). Five published RCTs included a general statement, for example, following international principles, applicable laws and regulations (Chen-2020a, Dougan-2021a, Dougan-2021b, Gupta-2021a, Podder-2020). The study authors of these eight published RCTs were contacted to confirm ethics approval. On request, one study author (Mareev-2021) confirmed ethics approval without providing further details (ie, EN).

**Table 1:** Reporting and identification of ethics details in RCTs

Ethics details	RCTs, n (%)	
	Journal/preprint articles (n=182)	Registration records (n=6)
**Statement on ethics approval**		
Reported in primary study report*	174 (96%)	1 (17%)
Insufficiently reported/not reported	8 (5/3) (4%)	5 (0/5) (83%)
Identified by author request	1	N/A
Not found/unclear	7	N/A
**Name and location ofEC**		
Reported in primary study report*	130 (71%)	1 (17%)
Insufficiently reported (generic statement/not reported)	52 (11/41) (29%)	5 (0/5) (83%)
Identified in the trial registration record	8	N/A
Identified by author request	12	N/A
Not found/unclear	32	N/A
**ECrecognised(n=151)**		
Yes	133	1
Unclear	17	0
Due to EC not found in the source	14	0
Due to EC not found, no source available	3	0
**Ethics approval number**		
Reported in primary study report*	81 (45%)	1 (17%)
Not reported	101 (55%)	5 (83%)
Identified in the trial registration record	25	N/A
Identified by author request	19	N/A
Not found	57	N/A
**Informed consent**		
Reported in the primary study report	165 (91%)	6 (100%)
Insufficiently reported/not reported	17 (14/3) (9%)	0
Clarified by author request	3	N/A
Not found/unclear	14	N/A

* Primary study report=publication/preprint or registration record, if RCT unpublished.

ECethics committeeN/Anot applicableRCTs, randomised controlled trials

Of 188 RCTs, 130 published and 1 unpublished RCT provided the name and location of at least one EC in the primary study report (table 1). 11 published RCTs included only a generic statement without further reference to the name and location of the EC, including 8 international multicentre RCTs. 41 published RCTs and 5 unpublished RCTs did not indicate the name and/or the location of at least one EC in the primary study report. Eight published RCTs provided this information exclusively in the trial registration record (Chen-2020b, Galan-2021, Huang-2020, Soin-2021, Spinner-2020, Thakar-2021). On request, 12 study authors supplied the name and location of ECs. In 37 RCTs, the EC remained unreported or unclear. Of 151 RCTs with reported or identified name and location of at least one EC, 134 were classified as recognised (table 1). The national recognition status of the ECs in the remaining 17 RCTs was unclear. For 14 RCTs, the EC could not be identified in any of our sources (online supplemental addtional file 1) and for the remaining 3 RCTs there was no source available (ie, two RCTs from Iraq: Hashim-2020, Rasheed-2020; one from Russia: Mareev-2021).

81 published RCTs and 1 unpublished RCT included the EN in the primary study report (table 1). 101 published RCTs and 5 unpublished RCTs did not report any EN in the primary study report. For 25 RCTs, the EN was identified in the trial registration records. For another 19 published RCTs, the study authors provided the EN on request. The EN was not found for 57 published RCTs.

Of 188 RCTs, 165 published RCTs and all 6 unpublished RCTs included a statement that IC was obtained (table 1); 163 obtained IC in written form and 2 RCTs obtained verbal or written IC which was approved by an EC (Dequin-2020, Derde-2021). In 14 RCTs, a statement on IC was reported; however, it was unclear if it was written consent or verbal, but approved by an EC. Three study authors confirmed having obtained written IC from all participants on request (Gonzalez-2021, Okumus-2021, van den Berg-2022). In three RCTs, the publication contained no statement on IC (Galan-2021, Li-2021, Mareev-2021).

Of the 188 RCTs, 41% sufficiently reported and fulfilled all items required for the assessment of the ethics approval in the primary study report and/or the trial registration record, that is, 77 published RCTs and 1 unpublished RCT (table 2). We classified these RCTs as ‘no concern’. After the first assessment round, 105 published RCTs and 5 unpublished RCTs incompletely reported at least one item required to assess the ethics approval domain and were categorised as ‘awaiting classification’. None of the RCTs were excluded since there was no RCT with a confirmed lack of an ethics approval or IC. Of the 110 RCTs categorised as ‘awaiting classification’, 95 study authors were contacted due to missing information regarding statement of ethics approval, EC, EN and/or IC (table 2). Study authors of 25 RCTs responded and 21 RCTs were finally upgraded to ‘no concern’. One study author provided sufficient information; however, the EC was not found in our sources (ie, unclear national recognition status) and the RCT remained ‘awaiting classification’ (Sadeghipour-2021). Three RCTs remained ‘awaiting classification’ due to incomplete or insufficient responses (Abd-Elsalam-2021a, Ali-2022, Mareev-2021) and study authors of 70 RCTs did not respond at all. 15 study authors were not contacted, which included 5 unpublished RCTs and 10 RCTs providing complete information in the primary study reports; however, the EC could either not be found in our sources, or there was no source available to verify the recognition status of the ECs (table 2). Following the author request, a total of 99 RCTs were considered as ‘no concern’ and 89 RCTs remained as ‘awaiting classification’.

**Table 2:** Summary of research integrity assessment based on provided ethics information

Information on ethics	No concern	Awaiting classification	Exclude
**Reporting of information (n=188)**			
Information completely reported in publication and/or in registration record(s)	78	0	N/A
Journal/preprint publication	77	0	N/A
Registration records	1	0	N/A
Information incompletely reported in publication and/or in registration record(s)	0	110	N/A
Journal/preprint publication	0	105	N/A
Registration records	0	5	N/A
**Retrieval of information of** randomised controlled trials **‘awaiting classification’ (n=110) (ie, author request)**			
Study author contacted for missing information or inconsistencies:	21	74	N/A
Information completed by study author	21	1*	N/A
Response insufficient or not complete	0	3	N/A
No response received	0	70	N/A
Study author not contacted for following reasons:	0	15	N/A
Only registration record available	0	5	N/A
Information complete, but EC not found in source	0	8	N/A
Information complete, but no source for national ECs available	0	2	N/A
**Final assessment**	99	89	0

* Sadeghipour-2021 (INSPIRATION) provided complete information, but EC was not found in source (national recognition unclear).

EC, ethics committeeN/Anot applicable

We have differentiated the reasons for ‘awaiting classification’ in reporting-related and RI-related reasons (table 3). Several RCTs comprised more than one reason for ‘awaiting classification’ of both types, that is, RI-related and reporting-related. Most RCTs were ‘awaiting classification’ due to reporting-related reasons. Predominantly, an EN was not reported in 62 RCTs, followed by insufficient or not reported EC in 37 RCTs. 17 cases of RI-related reasons were counted which are not due to non-reporting, but to a lack of EC recognition.

**Table 3:** Reasons for awaiting classification

Reasons for awaiting classification (n=89), after author request with or without response	
**Reporting-related reasons** *	
Statement on ethics approval insufficient or not reported	12†
EC insufficient or not reported	37†
EN not reported	62†
IC unclear or not reported	14
**RI-related reasons**	
EC reported, but national recognition unclear	17
EC not found in source	14
No source available	3

* Some RCTs have more than one reason for ‘awaiting classification’;, therefore, the sum of all reasons exceeds the total number of RCTs in ‘awaiting classification’.

† Including five RCTs available as registration records only.

EC**,** ethics committee; EN, ethics approval number; IC, informed consentRCTsrandomised controlled trialsRI, research integrity

In this study, we extracted and assessed the date of ethics approval (ie, date of ethics opinion) in addition to the original five RIA domains. Five published RCTs (Chowdhury-2021, Gonzalez-2021, Thakar-2021, Vallejos-2021, van den Berg-2022) and one unpublished RCT (NCT04392141) reported the date of ethics approval in the primary study report, and in all cases the ethics approval had been obtained prior to study start (table 4). Six published RCTs (Bennett-Guerrero-2021, Chen-2021, Faisal-2021, Hamdy Salman-2020, Okumus-2021, Rasheed-2020) included a general statement (eg, ethics approval obtained prior to enrolment), without specifying the date of ethics approval. 171 published and 5 unpublished RCTs did not provide the ethics approval date in the primary study report. For 74 of the 177 published RCTs, the ethics approval date could be identified in one or more trial registration record, that is, 50 registrations in the EU Clinical Trials Register (EUCTR), 15 in the International Standard Randomised Controlled Trial Number (ISRCTN), 12 in the Iranian Registry of Clinical Trials (IRCT), 7 in the Chinese Clinical Study Register (ChiCTR) and 2 in the Clinical Trials Registry India (CTRI) (online supplemental additional file 4). In total, the ethics approval date for 103 published RCTs could not be retrieved despite active search. Of 80 RCTs with an identified ethics approval date, 63 RCTs had obtained the ethics approval prior to study start/enrolment of the first subject for at least one site, including 15 single-centre RCTs, 34 national multicentre and 14 international multicentre RCTs. 14 RCTs presented inconsistencies in the study start dates reported in the primary study reports and the trial registration records, resulting in different assessments of ethics approval timing. Of these 14 RCTs with inconsistencies, 6 were single-centre (Babalola-2022, Chen-2020b, Farahani-2020, Huang-2020, Kharazmi-2022, Pouladzadeh-2021), 2 were national multicentre RCTs (Corral-Gudino-2021, Wang-2020a) and the remaining 6 RCTs were international multicentre RCTs (Eom-2021, Gupta-2021a, Gupta-2021b, Rosas-2021a, Spinner-2020, Tardif-2021), in which the primary study location remained unclear (ie, possibly different date of EA). Two published national multicentre RCTs from China (Li-2020, Li-2021) and one international multicentre RCT (Pan-2020) received an ethics approval after study start and were considered ‘potentially retrospective’ according to the publicly available information from the trial registries. The study authors did not provide any feedback on request, leaving us unable to clarify the dates.

**Table 4:** Ethics approval date of RCTs

Ethics approval date (ie, date of ethics opinion)	RCTs, n (%)	
	Journal/preprint articles (n=182)	Registration records (n=6)
**Reporting of ethics approval date**		
Reported in primary study report*	5 (3%)	1 (17%)
Insufficiently reported/not reported	177 (6/171) (97%)	5 (83%)
Identified in the trial registration record	74	N/A
Not found/unclear	103	N/A
**Ethics approval obtained prior to study start (n=80)**		
Yes†	62	1
Unclear	14	0
No	3	0

* Primary study report=publication/preprint or registration record, if RCT unpublished.

† For at least one site.

N/Anot applicableRCTs, randomised controlled trials

The frequency of reporting of the ethics approval date was similar in RCTs assessed as ‘no concern’ with 45% (45 of 99) and ‘awaiting classification’ with 39% (35 of 89). However, two of three RCTs with ‘potentially retrospective’ ethics approval (Li-2020, Li-2021) were assessed as ‘awaiting classification’ while the other was assessed as ‘no concern’ (Pan-2020).

The comparison of RCTs with sufficiently reported ethics approval regarding all items (ie, ‘no concern’, n=99) compared with those with insufficient, inconsistent or missing ethics approval details (ie, ‘awaiting classification’, n=89) revealed a similar distribution regarding the study setting, that is, international multicentre (18% vs 16%), national multicentre (47% vs 49%) and single-centre (35% vs 35%) (table 5). According to the countries of study conduct, 66% of the European RCTs were assessed as ‘no concern’, whereas 71% of the North American RCTs were ‘awaiting classification’ (table 5). Half of all single-centre RCTs were from Asia, with 58% assessed as ‘awaiting classification’.

**Table 5:** Characteristics of RCTs classified as ‘no concern’ and ‘awaiting classification’ (n=188)

Study characteristics	No concern(n=99)	Awaiting classification (n=89)
**Setting and location**		
Multicentre, international (n=32)	18	14
Multicentre, national (n=90)	46	44
Africa	1	0
Asia	10	12
Australia	0	0
Europe	22	14
North America	5	13
South America	8	5
Single-centre (n=66)	35	31
Africa	6	3
Asia	14	19
Australia	0	0
Europe	5	0
North America	2	4
South America	8	5
Trial registry*		
ClinicalTrials.gov (n=142)	76	66
EUCTR (n=56)	35	21
ISRCTN (n=15)	15	0
IRCT (n=12)	7	5
ChiCTR (n=9)	0	9
CTRI (n=6)	4	2
ReBec (n=5)	4	1
Other† (n=4)	3	1
RCTs without trial registration (n=12)	4	8
**Sample size;randomisedpatients**		
Median (IQR)	240 (91 to 777)	131 (65 to 420)
Less than 100 participants (n=60)	26	34
100 to less than 200 participants (n=40)	20	20
200 or more participants (n=88)	53	35

* Some RCTs were registered in different trial registries and/or with different numbers in the same registry; therefore, the sum of all registrations exceeds the total number of RCTs in the categories ‘no concern’ and ‘awaiting classification’.Includes two registrations in REec, one in INA, and one in SCTR.

† Includes two registrations in REec, one in INA, and one in SCTR.Some RCTs were registered in different trial registries and/or with different numbers in the same registry, therefore, the sum of all registrations exceeds the total number of RCTs in the categories no concern and awaiting classification.

ChiCTR, Chinese Clinical Study Register; CTRI, Clinical Trials Registry India; EUCTR, EU Clinical Trials Register; INA, Indonesia Clinical Research Registry; IRCT, Iranian Registry of Clinical Trials; ISRCTN, International Standard Randomised Controlled Trial Number; RCTs, randomised controlled trials; ReBec, Brazilian Registry of Clinical Trials; REec, Spanish Clinical Study Registry [Registro Español de Estudios Clínicos]; SCTR, Saudi Clinical Study Registry

12 RCTs were not registered, and 8 of them were assessed as ‘awaiting classification’ for the RIA domain on ethics approval (table 5). ClinicalTrials.gov was the most used platform for trial registration among the registered RCTs, with no difference in the number of registrations between RCTs assessed as ‘no concern’ compared with ‘awaiting classification’ (76 vs 66). We identified nine registrations on ChiCTR and all nine belonged to RCTs which were assessed as ‘awaiting classification’. In contrast, all 15 registrations in the WHO ISRCTN registry belonged to RCTs assessed as ‘no concern’.

There was a slight difference between RCTs categorised as ‘no concern’ compared with ‘awaiting classification’ in terms of the sample sizes, that is, 54% versus 39% randomised 200 or more participants, and the median sample size was 240 versus 131 participants.

## Discussion

Reporting of ethics approval in COVID-19 RCTs is insufficient. Only 41% of RCTs sufficiently reported and fulfilled all items required for the assessment of ethics approval in the primary study report and/or the trial registration record, whereas 59% of RCTs incompletely reported ethics items and were categorised as ‘awaiting classification’. In our study, registration records have proven to be important sources for ethics approval details as we were able to identify details for 33 items. Furthermore, authors of 50% of included RCTs were contacted due to missing information or to clarify inconsistencies. This enabled an upgrade of another 11% of the RCTs from ‘awaiting classification’ to ‘no concern’. Finally, almost half of all RCTs were considered of ‘no concern’ and the other half remained ‘awaiting classification’, that is, not eligible for evidence synthesis due to uncertainties regarding ethics approval according to the RIA tool.

Originally, we planned to assess RCTs with confirmed lack of an ethics approval or lack of IC as ‘exclude’, that is, not eligible for evidence synthesis as problematic in terms of RI. About 10% of RCTs in our study pool did not sufficiently report on IC. However, in no case were we clearly able to demonstrate that ethics approval or IC was not present. A main obstacle to a reliable assessment is that we cannot determine with certainty whether there was actually a violation of RI in terms of obtaining ethics approval and IC for a clinical study that goes beyond the problem of non-reporting.

The reporting of ethics approval in clinical studies has improved little over the years. Zoccatelli *et al* determined that 97% of the studies published in 2011 in the *European Journal of Anaesthesiology* declared an ethics approval, of which 85% reported the name and location of EC and 69% provided EN.^15^ Yank *et al* estimated a lack of ethics approval statement in 18% of the studies carried out after 1997, and a tenth of the RCTs did not report having obtained written IC.^16^ A hurdle might be that reporting of ethics approval in RCTs is currently not included as an item in the CONSORT 2010 checklist, the most important reporting guideline for RCTs.^11^ We assume that reporting could be improved if CONSORT would include items on ethics approval due to its major impact on reporting quality of RCTs in the past, as shown for the registration of studies.^17^ An update of the CONSORT 2010 checklist is currently in progress, and we expect consideration of ethics approval as ECs and regulatory agencies will be important stakeholders.^18^ The current situation requires that we first increase awareness of the importance of reporting ethics approval in study reports before producers of evidence syntheses can reliably assess this aspect in the context of RI.

Our post hoc comparison of RCTs assessed as ‘no concern’ with those assessed as ‘awaiting classification’ revealed a similar distribution regarding study setting and country, and only a slight difference regarding the median sample size. The fact that the large international multicentre studies behaved similarly in the reporting of ethics approval as the small single-centre studies could be another indication of the lack of awareness of the importance of full reporting.

Our study outlines the essential role trial registries play in the assessment of ethics approval. Even though most trial registries, especially those listed as ‘primary registries in the WHO network’, explicitly require proof of ethics approval prior to trial registration, our study reveals that several registries do not always enforce these requirements. This is especially true for ChiCTR as none of the seven RCTs registered with nine registrations on this platform were considered ‘no concern’ due to inconsistencies or non-reporting of ethics details. Among the registries used by our RCTs, ISRCTN, ChiCTR, EUCTR, CTRI and IRCT provided detailed information pertaining to ethics approval as a standard feature. Other platforms, such as ClinicalTrials.gov, where most of the RCTs were registered, do not supply information related to ethics approval as standard.

While the author response request has been established as another practical way to obtain missing information or clarify inconsistencies, this method is time consuming and was not effective in about three-quarters of all requests. Whether this effort can be carried out as part of an evidence synthesis mainly depends on the available resources and considerations on time and timeliness in individual cases. However, improved reporting would significantly reduce this time expenditure.

We have underpinned our assurance of adequate ethics approval through the national recognition of the ECs. The unavailability of updated national or international directories for accredited ECs/IRBs hinders our assessment. The search for national sources was time-consuming. About 10% of the RCTs were approved by an EC, which, after an extensive search, could not be found in any source, and therefore their recognition status remains unclear. The language barrier might play an important role in the search for sources. This finding differs from those of Zoccatelli *et al*, who were unable to identify 41% of the ECs of their study pool.^15^ On the other hand, our sources used for the assessment of the recognition status might be out of date: the EC might have been deactivated or lost national recognition status by the time of the approval provided to the study authors, or the committee may have lost but then regained national recognition status by the time of our search. A regularly updated international register of the recognised EC and IRBs would support the assessment of ethics in evidence synthesis.

One of the included studies, a large multicentre RCT (Pan-2020), used an online GCP-compliant system for data collection across many collaborating sites and countries. The designers of such systems could lock data entry until the EC grants a favourable opinion and authorises unlocking for the initiation of study recruitment. Simple technological solutions such as this could help harmonise new technologies with older international standards.^9^

Our study has several limitations. Our assessment is dependent on details reported in study reports. As stated by Tramèr^19^ and Yank *et al*,^16^ even when details pertaining to ethics approval are reported or provided by the author, there are no guarantees as to whether the approved protocol matches the study performed. Furthermore, we did not contact the EC to verify whether the authors obtained ethics approval, the information provided was consistent with the approval documents, or the EC was nationally recognised at the time of approval. We did not take into account the role of the journals in the assessment of this domain, although they may act as a checkpoint to screen ethically unsound studies and there may be great differences between different journals, as stated by Myles *et al*.^20^ Our search was conducted exclusively in English, which may hamper the retrieval of sources of EC in non-native English-speaking countries.

Finally, we need an extensive discussion in the research community on which ethical items should be used for a reliable assessment. The ongoing INSPECT-SR project is developing a tool to identify problematic RCTs in systematic reviews of healthcare-related interventions by critical discussion with different experts in the field on which checks are useful to determine a study’s authenticity, and ethics is one of the checks.^21^ Although currently not an item of the RIA ethics approval domain, we suggest the assessment of the ethics approval date as a new item.

In our view, it is essential to include ethics approval in the RIA of RCTs as part of evidence synthesis in the future. However, poor reporting—even if a RI issue on its own—complicates a reliable assessment. Nevertheless, including ethics approval in the checklist of evidence syntheses and in reporting guidelines of clinical studies is especially important to increase awareness in the scientific community about the need for high ethical standards in human research.^22^ Therefore, at present, we suggest to assess ethics approval in RCTs during a RIA included in the evidence synthesis process; however, not to exclude studies assessed as ‘awaiting classification’ from the study pool and meta-analysis, but instead perform sensitivity analysis to investigate the robustness of the results.

## Conclusion

This study highlights two main issues concerning ethics approval in RCTs. First, reporting of ethical approval in RCTs is poor. Second, as under-reporting cannot be excluded, the assessment of ethics as part of the RIA tool is currently not reliable. The lack of standardised procedures and guidelines for reporting information regarding ethics approval in the primary study record impedes the assessment, and therefore, leaves producers of evidence synthesis with a high number of RCTs which are not (completely) eligible for evidence synthesis (ie, RCTs categorised as ‘awaiting classification’). Updated reporting guidelines may improve this issue. Furthermore, we determined that trial registries play an essential role as providers of information pertaining to ethics approval and as sentinels, ensuring that RCTs are carried out according to available ethics regulations.

## supplementary material

10.1136/bmjopen-2024-092244 online supplemental file 1

10.1136/bmjopen-2024-092244 online supplemental file 2

10.1136/bmjopen-2024-092244 online supplemental file 3

10.1136/bmjopen-2024-092244 online supplemental file 4

## Data Availability

Datasets are included in this published article, supplementary information files, or available from the OSF repository, DOI: 10.17605/OSF.IO/9VZME.

## References

[1] Sterne JAC, Savović J, Page MJ (2019). RoB 2: a revised tool for assessing risk of bias in randomised trials. BMJ.

[2] Balshem H, Helfand M, Schünemann HJ (2011). GRADE guidelines: 3. Rating the quality of evidence. J Clin Epidemiol.

[3] Wilkinson J, Heal C, Antoniou GA (2024). Protocol for the development of a tool (INSPECT-SR) to identify problematic randomised controlled trials in systematic reviews of health interventions. BMJ Open.

[4] Mol BW, Lai S, Rahim A (2023). Checklist to assess Trustworthiness in RAndomised Controlled Trials (TRACT checklist): concept proposal and pilot. Res Integr Peer Rev.

[5] Weeks J, Cuthbert A, Alfirevic Z (2023). Trustworthiness assessment as an inclusion criterion for systematic reviews—What is the impact on results?. Cochrane Evidence Synthesis and Methods.

[6] Mousa A, Flanagan M, Tay CT (2024). Research Integrity in Guidelines and evIDence synthesis (RIGID): a framework for assessing research integrity in guideline development and evidence synthesis. EClinicalMedicine.

[7] NIH What is research integrity. https://grants.nih.gov/policy-and-compliance/policy-topics/research-integrity.

[8] EMA Glossary - regulatory terms “good clinical practice”. https://www.ema.europa.eu/ro/taxonomy/term/43145.

[9] WMA Declaration of Helsinki – ethical principles for medical research involving human participants. https://www.wma.net/policies-post/wma-declaration-of-helsinki/.

[10] Kolstoe SE, Pugh J (2024). The trinity of good research: Distinguishing between research integrity, ethics, and governance. Account Res.

[11] Schulz KF, Altman DG, Moher D (2010). CONSORT 2010 statement: updated guidelines for reporting parallel group randomised trials. BMJ.

[12] Weibel S, Popp M, Reis S (2023). Identifying and managing problematic trials: A research integrity assessment tool for randomized controlled trials in evidence synthesis. Res Synth Methods.

[13] International Council for Harmonisation of Technical Requirements for Pharmaceuticals for Human Use (ICH) https://database.ich.org/sites/default/files/E6_R2_Addendum.pdf.

[14] Weibel S (2024). Data from: investigation of ethics as part of a research integrity assessment of randomized controlled trials in covid-19 evidence syntheses.

[15] Zoccatelli D, Tramèr MR, Elia N (2018). Identification of ethics committees based on authors’ disclosures: cross-sectional study of articles published in the European Journal of Anaesthesiology and a survey of ethics committees. BMC Med Ethics.

[16] Yank V, Rennie D (2002). Reporting of informed consent and ethics committee approval in clinical trials. JAMA.

[17] Lamberink HJ, Vinkers CH, Lancee M (2022). Clinical Trial Registration Patterns and Changes in Primary Outcomes of Randomized Clinical Trials From 2002 to 2017. JAMA Intern Med.

[18] Hopewell S, Boutron I, Chan A-W (2022). An update to SPIRIT and CONSORT reporting guidelines to enhance transparency in randomized trials. Nat Med.

[19] Tramèr MR (2012). Ethical requirements and authorship: not much room for interpretation. Eur J Anaesthesiol.

[20] Myles PS, Tan N (2003). Reporting of ethical approval and informed consent in clinical research published in leading anesthesia journals. Anesthesiology.

[21] Wilkinson J, Heal C, Antoniou GA (2024). A survey of experts to identify methods to detect problematic studies: Stage 1 of the INSPECT-SR Project. medRxiv.

[22] Weingarten MA, Paul M, Leibovici L (2004). Assessing ethics of trials in systematic reviews. BMJ.

