# Supplementary material for: Investigation of ethics approval as part of a research integrity assessment of randomised controlled trials in COVID-19 evidence syntheses: a meta-epidemiological study
Source: BMJ Open. 2025 Mar 24;15(3):e092244. doi: 10.1136/bmjopen-2024-092244 (PMC11934354; doi:10.1136/bmjopen-2024-092244)
Supplement: online supplemental file 1 [file bmjopen-15-3-s001.docx]

**Additional File 1: Directory of consulted sources for ethics committees, last edited on 27.07.2023**

| **Country** | **Source provided by a national organization** | **Source provided by an international organization** | **Institutional source** | **Notes** |
| --- | --- | --- | --- | --- |
| **Argentina** | https://www.argentina.gob.ar/salud/investigaciones/comites | Not found | https://med.unne.edu.ar/institucional/gestion-academica-y-administrativa/secretaria-de-ciencia-y-tecnologia/comite-de-bioetica-en-investigacion-de-ciencias-de-la-salud/ |  |
|  | https://buenosaires.gob.ar/sites/default/files/2023-09/Lista_nueva_cei_efectores_no_gcba_4-sep-2023.pdf |  |  |  |
|  | https://www.argentina.gob.ar/sites/default/files/2022/06/listado_c  omites_acreditados_8-7-2022.pdf |  |  |  |
| **Bahrain** | Not found | Not found | https://www.rcsi.com/bahrain/research/research-ethics-committee |  |
| **Bangladesh** | Not found | https://healthresearchwebafrica.org.za/en/bangladesh/institution | Not found |  |
| **Belgium** | https://www.famhp.be/sites/default/files/Lijst%20EC's%20-%20Liste%20CE_1.pdf | Not found | Not found |  |
| **Brazil** | https://plataformabrasil.saude.gov.br/login.jsf | Not found | Not found | CONEP= Brazilian committee of ethics in Human Research |
| **Chile** | Not found | Not found | https://eticayseguridad.uc.cl/images/DR._143-2020._Regulation_of_the_scientific_ethics_committee_of_health_UC_-_English.pdf |  |
| **China** | Not found | https://www.aahrpp.org/find-an-accredited-organization | Not found |  |
| **Colombia** | https://aciccolombia.org/lista-de-comites-de-etica-en-colombia/ | https://healthresearchwebafrica.org.za/en/colombia/institution | Not found | National source not used |
| **Ecuador** | Not found | Not found | https://www.usfq.edu.ec/en/human-research-ethics-committee-ceish |  |
| **Egypt** | http://www.enrec.org/directory | Not found | Not found |  |
| **France** | http://urcest.com/les-cpp-en-ile-de-france | Not found | Not found |  |
|  | http://cppouest1.fr/mediawiki/index.php?title=Accueil |  |  |  |
| **Germany** | https://www.bfarm.de/DE/Medizinprodukte/Ueberblick/Institutionen/Ethikkommissionen/_node.html | Not found | Not found |  |
| **Greece** | Not found | http://www.eurecnet.org/information/greece.html | Not found |  |
| **India** | https://cdsco.gov.in/opencms/export/sites/CDSCO_WEB/Pdf-documents/list-ofecommittee2022.pdf | Not found | Not found |  |
|  | https://cdsco.gov.in/opencms/opencms/en/Clinical-Trial/Ethics-Committee/Ethics-Committee-Re-Registration/ |  |  |  |
|  | https://www.ncdirindia.org/All_Committee.html |  |  |  |
| **Indonesia** | Not found | https://www.ncdirindia.org/All_Committee.html | Not found | official website of National Institute of Health Research and Development on 07.02.24 not available, https://health-policy-systems.biomedcentral.com/articles/10.1186/s12961-015-0024-9 |
| **Iran** | Not found | https://healthresearchwebafrica.org.za/en/iran/institution | Not found |  |
| **Iraq** | Not found | Not found | Not found | Source not found |
| **Israel** | Not found | Not found | https://rnd.sheba.co.il/Research_Authority | Link no longer available (last visited 07.2023) |
| **Italy** | https://www.aifa.gov.it/documents/20142/1123276/Comunicazione_gestione_studi_clinici_in_emergenza_COVID-19-EN_17.09.2020.pdf | Not found | Not found |  |
| **Mexico** | https://www.gob.mx/cms/uploads/attachment/file/801449/Registros_CEI.01022023.pdf | Not found | Not found |  |
| **Nigeria** | https://nhrec.net/registered-health-research-ethics-committees-in-nigeria-hrec/ | Not found | Not found |  |
| **Norway** | https://rekportalen.no/#omrek/REK | Not found | Not found |  |
| **Oman** | Not found | Not found | https://royalhospital.med.om/pdf/RH-Research-Policy.pdf |  |
| **Pakistan** | nbcpakistan.org.pk/assets/list_of_ercin_publicprivatemedicalcolleges.docx | Not found | Not found |  |
| **Qatar** | Not found | Not found | https://www.hamad.qa/EN/About-Us/Our-Accreditations/Pages/default.aspx# |  |
| **Russia** | Not found | Not found | Not found | Source not found |
| **Saudi Arabia** | https://www.moh.gov.sa/en/Ministry/MediaCenter/Publications/Pages/Annual-Research-Report-2016.pdf | Not found | Not found |  |
| **Singapore** | Not found | https://www.aahrpp.org/find-an-accredited-organization | Not found |  |
| **South Africa** | https://www.health.gov.za/wp-content/uploads/2022/05/NHREC-Registration-List-of-Human-RECs-Animal-RECs-registered-with-NHREC.pdf | Not found | Not found |  |
| **South Korea** | Not found | https://www.aahrpp.org/find-an-accredited-organization | Not found |  |
| **Spain** | https://www.aemps.gob.es/medicamentos-de-uso-humano/investigacion_medicamentos/investigacionclinica_ceim/ | Not found | Not found |  |
| **Sweden** | https://etikprovningsmyndigheten.se/en/ | Not found | Not found |  |
| **Taiwan** | https://www.taiwanclinicaltrials.tw/spotlight/clinical_trial_overview/c_IRB/introduce | Not found | Not found |  |
| **The Netherlands** | https://english.ccmo.nl/mrecs/accredited-mrecs | Not found | Not found |  |
| **Turkey** | https://www.titck.gov.tr/dinamikmodul/84 | Not found | Not found |  |
| **Uganda** | Not found | https://healthresearchwebafrica.org.za/en/uganda/institution | Not found |  |
| **UK** | https://www.hra.nhs.uk/about-us/committees-and-services/res-and-recs/search-research-ethics-committees/ | Not found | Not found |  |
| **USA** | https://ohrp.cit.nih.gov/search/irbsearch.aspx | Not found | Not found |  |
| **Multisite** | N/A | https://www.who.int/groups/research-ethics-review-committee | N/A | WHO ethics review committee |
