# Supplementary material for: Investigation of ethics approval as part of a research integrity assessment of randomised controlled trials in COVID-19 evidence syntheses: a meta-epidemiological study
Source: BMJ Open. 2025 Mar 24;15(3):e092244. doi: 10.1136/bmjopen-2024-092244 (PMC11934354; doi:10.1136/bmjopen-2024-092244)
Supplement: online supplemental file 2 [file bmjopen-15-3-s002.docx]

**Additional File 2**: References to included Cochrane reviews and systematic reviews

We selected 23 evidence syntheses with the largest RCT pool in our search on 13 different interventions for prevention or treatment of COVID-19^1-23^; 13 were Cochrane reviews^2 3 5-7 11 13 15-17 19-21^ and ten were non-Cochrane systematic reviews^1 4 8-10 12 14 18 22 23^. Three systematic reviews investigated two different interventions with the largest RCT pool in our search, i.e. Deng-2022 (convalescent plasma and SARS-CoV-2-neutralising monoclonal antibodies)^4^, Siemieniuk-2020 (hydroxychloroquine or chloroquine and systemic corticosteroids)^18^, and Zhang-2021(antibiotics and inhaled corticosteroids)^23^.

1. Aamir Waheed M, Rashid K, Rajab T, et al. Role of anticoagulation in lowering the mortality in hospitalized covid-19 patients: Meta-analysis of available literature. *Saudi Med J* 2022;43(6):541-50. doi: 10.15537/smj.2022.43.6.20220046

2. Ansems K, Grundeis F, Dahms K, et al. Remdesivir for the treatment of COVID-19. *Cochrane Database Syst Rev* 2021;8(8):Cd014962. doi: 10.1002/14651858.Cd014962

3. Davidson M, Menon S, Chaimani A, et al. Interleukin-1 blocking agents for treating COVID-19. *Cochrane Database Syst Rev* 2022;1(1):Cd015308. doi: 10.1002/14651858.Cd015308

4. Deng J, Heybati K, Ramaraju HB, et al. Differential efficacy and safety of anti-SARS-CoV-2 antibody therapies for the management of COVID-19: a systematic review and network meta-analysis. *Infection* 2022:1-15. doi: 10.1007/s15010-022-01825-8

5. Flumignan RL, Civile VT, Tinôco JDS, et al. Anticoagulants for people hospitalised with COVID-19. *Cochrane Database Syst Rev* 2022;3(3):Cd013739. doi: 10.1002/14651858.CD013739.pub2

6. Ghosn L, Chaimani A, Evrenoglou T, et al. Interleukin-6 blocking agents for treating COVID-19: a living systematic review. *Cochrane Database Syst Rev* 2021;3(3):Cd013881. doi: 10.1002/14651858.Cd013881

7. Griesel M, Wagner C, Mikolajewska A, et al. Inhaled corticosteroids for the treatment of COVID-19. *Cochrane Database Syst Rev* 2022;3(3):Cd015125. doi: 10.1002/14651858.Cd015125

8. Hosseini B, El Abd A, Ducharme FM. Effects of Vitamin D Supplementation on COVID-19 Related Outcomes: A Systematic Review and Meta-Analysis. *Nutrients* 2022;14(10) doi: 10.3390/nu14102134

9. Izcovich A, Peiris S, Ragusa M, et al. Bias as a source of inconsistency in ivermectin trials for COVID-19: A systematic review. Ivermectin's suggested benefits are mainly based on potentially biased results. *J Clin Epidemiol* 2022;144:43-55. doi: 10.1016/j.jclinepi.2021.12.018

10. Kow CS, Lee LH, Ramachandram DS, et al. The effect of colchicine on mortality outcome and duration of hospital stay in patients with COVID-19: A meta-analysis of randomized trials. *Immun Inflamm Dis* 2022;10(2):255-64. doi: 10.1002/iid3.562

11. Kreuzberger N, Hirsch C, Chai KL, et al. SARS-CoV-2-neutralising monoclonal antibodies for treatment of COVID-19. *Cochrane Database Syst Rev* 2021;9(9):Cd013825. doi: 10.1002/14651858.CD013825.pub2

12. Lee TC, Murthy S, Del Corpo O, et al. Remdesivir for the treatment of COVID-19: A systematic review and meta-analysis. *Clin Microbiol Infect* 2022 doi: 10.1016/j.cmi.2022.04.018

13. Mikolajewska A, Fischer AL, Piechotta V, et al. Colchicine for the treatment of COVID-19. *Cochrane Database Syst Rev* 2021;10(10):Cd015045. doi: 10.1002/14651858.Cd015045

14. Naveed Z, Sarwar M, Ali Z, et al. Anakinra treatment efficacy in reduction of inflammatory biomarkers in COVID-19 patients: A meta-analysis. *J Clin Lab Anal* 2022;36(6):e24434. doi: 10.1002/jcla.24434

15. Piechotta V, Iannizzi C, Chai KL, et al. Convalescent plasma or hyperimmune immunoglobulin for people with COVID-19: a living systematic review. *Cochrane Database Syst Rev* 2021;5(5):Cd013600. doi: 10.1002/14651858.CD013600.pub4

16. Popp M, Stegemann M, Metzendorf MI, et al. Ivermectin for preventing and treating COVID-19. *Cochrane Database Syst Rev* 2021;7(7):Cd015017. doi: 10.1002/14651858.CD015017.pub2

17. Popp M, Stegemann M, Riemer M, et al. Antibiotics for the treatment of COVID-19. *Cochrane Database Syst Rev* 2021;10(10):Cd015025. doi: 10.1002/14651858.Cd015025

18. Siemieniuk RA, Bartoszko JJ, Ge L, et al. Drug treatments for covid-19: living systematic review and network meta-analysis. *Bmj* 2020;370:m2980. doi: 10.1136/bmj.m2980

19. Singh B, Ryan H, Kredo T, et al. Chloroquine or hydroxychloroquine for prevention and treatment of COVID-19. *Cochrane Database Syst Rev* 2021;2(2):Cd013587. doi: 10.1002/14651858.CD013587.pub2

20. Stroehlein JK, Wallqvist J, Iannizzi C, et al. Vitamin D supplementation for the treatment of COVID-19: a living systematic review. *Cochrane Database Syst Rev* 2021;5(5):Cd015043. doi: 10.1002/14651858.Cd015043

21. Wagner C, Griesel M, Mikolajewska A, et al. Systemic corticosteroids for the treatment of COVID-19. *Cochrane Database Syst Rev* 2021;8(8):Cd014963. doi: 10.1002/14651858.Cd014963

22. Yu SY, Koh DH, Choi M, et al. Clinical efficacy and safety of interleukin-6 receptor antagonists (tocilizumab and sarilumab) in patients with COVID-19: a systematic review and meta-analysis. *Emerg Microbes Infect* 2022;11(1):1154-65. doi: 10.1080/22221751.2022.2059405

23. Zhang C, Jin H, Wen YF, et al. Efficacy of COVID-19 Treatments: A Bayesian Network Meta-Analysis of Randomized Controlled Trials. *Front Public Health* 2021;9:729559. doi: 10.3389/fpubh.2021.729559
