# Supplementary material for: Investigation of ethics approval as part of a research integrity assessment of randomised controlled trials in COVID-19 evidence syntheses: a meta-epidemiological study
Source: BMJ Open. 2025 Mar 24;15(3):e092244. doi: 10.1136/bmjopen-2024-092244 (PMC11934354; doi:10.1136/bmjopen-2024-092244)
Supplement: online supplemental file 3 [file bmjopen-15-3-s003.pdf]

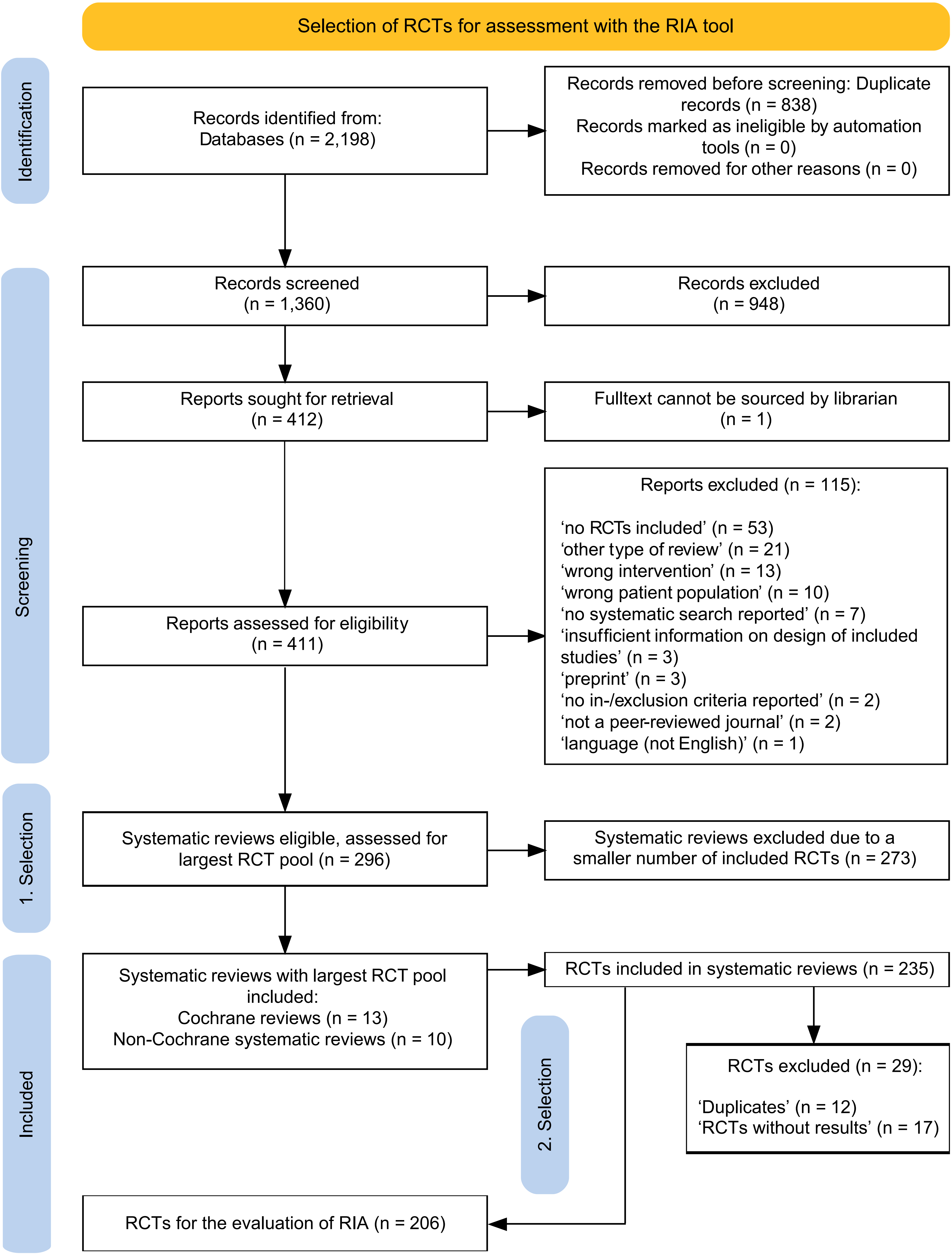

Selection of RCTs for assessment with the RIA tool
Identification
Records identified from:
Databases (n = 2,198)
Records removed before screening: Duplicate records (n = 838)
Records marked as ineligible by automation tools (n = 0)
Records removed for other reasons (n = 0)
Screening
Records screened
(n = 1,360)
Records excluded
(n = 948)
Reports sought for retrieval
(n = 412)
Fulltext cannot be sourced by librarian
(n = 1)
Reports assessed for eligibility
(n = 411)
Reports excluded (n = 115):
'no RCTs included' (n = 53)
'other type of review' (n = 21)
'wrong intervention' (n = 13)
'wrong patient population' (n = 10)
'no systematic search reported' (n = 7)
'insufficient information on design of included studies' (n = 3)
'preprint' (n = 3)
'no in-/exclusion criteria reported' (n = 2)
'not a peer-reviewed journal' (n = 2)
'language (not English)' (n = 1)
1. Selection
Systematic reviews eligible, assessed for largest RCT pool (n = 296)
Systematic reviews excluded due to a smaller number of included RCTs (n = 273)
Included
Systematic reviews with largest RCT pool included:
Cochrane reviews (n = 13)
Non-Cochrane systematic reviews (n = 10)
RCTs included in systematic reviews (n = 235)
2. Selection
RCTs excluded (n = 29):
'Duplicates' (n = 12)
'RCTs without results' (n = 17)
RCTs for the evaluation of RIA (n = 206)
