# Supplementary material for: Investigation of ethics approval as part of a research integrity assessment of randomised controlled trials in COVID-19 evidence syntheses: a meta-epidemiological study
Source: BMJ Open. 2025 Mar 24;15(3):e092244. doi: 10.1136/bmjopen-2024-092244 (PMC11934354; doi:10.1136/bmjopen-2024-092244)
Supplement: online supplemental file 4 [file bmjopen-15-3-s004.docx]

**Additional File 4: Ethics approval date reported in trial registry records. (n = 74)^a^**

| **Published RCTs** | **Registered on** | | **EA Date identified on** | |
| --- | --- | --- | --- | --- |
| **ACTIV-3/TICO-2021b** | | NCT, EUCTR | | EUCTR |
| **Ader-2021** | | NCT, EUCTR | | EUCTR |
| **Ader-2022** | | NCT, EUCTR | | EUCTR |
| **Agarwal-2020** | | CTRI | | CTRI |
| **Babalola-2022** | | ISRCTN | | ISRCTN |
| **Baldeón-2022** | | ISRCTN | | ISRCTN |
| **Butler-2021 (PRINCIPLE)** | | ISRCTN, EUCTR | | ISRCTN, EUCTR |
| **Caricchio-2021** | | NCT, EUCTR | | EUCTR |
| **Chaccour-2021** | | NCT, EUCTR | | EUCTR |
| **Chen-2020b** | | ChiCTR | | ChiCTR |
| **Chen-2020c** | | ChiCTR | | ChiCTR |
| **CORIMUNO-2021** | | NCT, EUCTR | | EUCTR |
| **CORIMUNO-2022** | | NCT, EUCTR | | EUCTR |
| **Corral-Gudino-2021** | | EUCTR, REec | | EUCTR |
| **Davoodi-2020** | | IRCT | | IRCT |
| **Declercq-2021** | | NCT, EUCTR | | EUCTR |
| **Deftereos-2020** | | NCT, EUCTR | | EUCTR |
| **Dequin-2020** | | NCT, EUCTR | | EUCTR |
| **Dorward-2022 (PRINCIPLE)** | | ISRCTN, EUCTR | | ISRCTN, EUCTR |
| **Dubée-2020** | | NCT, EUCTR | | EUCTR |
| **Edalatifard-2020** | | IRCT | | IRCT |
| **Entrenas Castillo-2020** | | NCT, EUCTR | | EUCTR |
| **Eom-2021** | | NCT, EUCTR | | EUCTR |
| **Farahani-2020** | | IRCT | | IRCT |
| **Ghaderkhani-2020** | | IRCT | | IRCT |
| **Gupta-2021a, COMET-ICE** | | NCT, EUCTR | | EUCTR |
| **Gupta-2021b, COMET-ICE** | | NCT, EUCTR | | EUCTR |
| **Hermine-2021** | | NCT, EUCTR | | EUCTR |
| **Hinks-2021** | | NCT, EUCTR | | EUCTR |
| **Huang-2020** | | ChiCTR | | ChiCTR |
| **Jamaati-2021** | | IRCT | | IRCT |
| **Karakike-2021** | | NCT, EUCTR | | EUCTR |
| **Kharazmi-2022** | | IRCT | | IRCT |
| **Kirti-2021** | | CTRI | | CTRI |
| **Körper-2021** | | NCT, EUCTR | | EUCTR |
| **Kyriazopoulou-2021** | | NCT, EUCTR | | EUCTR |
| **Lescure-2021** | | NCT, EUCTR | | EUCTR |
| **Li-2020** | | ChiCTR | | ChiCTR |
| **Li-2021** | | ChiCTR (3x) | | ChiCTR |
| **Merchante-2022** | | NCT, EUCTR, REec | | EUCTR |
| **Mitjà-2020a** | | NCT, EUCTR | | EUCTR |
| **Mitjà-2020b** | | NCT, EUCTR | | EUCTR |
| **Mitjà-2020c** | | NCT, EUCTR | | EUCTR |
| **Niaee-2021** | | IRCT | | IRCT |
| **O'Brien-2022** | | NCT, EUCTR | | EUCTR |
| **Pan-2020 (SOLIDARITY)** | | NCT, ISRCT | | ISRCTN |
| **Pouladzadeh-2021** | | IRCT | | IRCT |
| **PRINCIPLE-2021** | | ISRCTN, EUCTR | | ISRCTN, EUCTR |
| **Ranjbar-2021** | | IRCT | | IRCT |
| **RECOVERY-2020a** | | ISRCTN, EUCTR, NCT | | ISRCTN, EUCTR |
| **RECOVERY-2020b** | | ISRCTN, EUCTR, NCT | | ISRCTN, EUCTR |
| **RECOVERY-2021a** | | ISRCTN, EUCTR, NCT | | ISRCTN, EUCTR |
| **RECOVERY-2021b** | | ISRCTN, EUCTR, NCT | | ISRCTN, EUCTR |
| **RECOVERY-2021c** | | ISRCTN, EUCTR, NCT | | ISRCTN, EUCTR |
| **RECOVERY-2021d** | | ISRCTN, EUCTR, NCT | | ISRCTN, EUCTR |
| **RECOVERY-2022** | | ISRCTN, EUCTR, NCT | | ISRCTN, EUCTR |
| **Rosas-2021a** | | NCT, EUCTR | | EUCTR |
| **Rosas-2021b** | | NCT, EUCTR | | EUCTR |
| **Salama-2021** | | NCT, EUCTR | | EUCTR |
| **Salehzadeh-2020** | | IRCT | | IRCT |
| **Salvarani-2021** | | NCT, EUCTR | | EUCTR |
| **Sancho-López-2021** | | EUCTR | | EUCTR |
| **Sekhavati-2020** | | IRCT | | IRCT |
| **Shahbaznejad-2021** | | IRCT | | IRCT |
| **Sivapalan-2021** | | NCT, EUCTR | | EUCTR |
| **Somersan-Karakaya-2022** | | NCT, EUCTR | | EUCTR |
| **Spinner-2020** | | ISRCTN, EUCTR, NCT | | ISRCTN, EUCTR |
| **Tang-2020** | | ChiCTR | | ChiCTR |
| **Tardif-2021** | | NCT, EUCTR | | EUCTR |
| **Wang-2020a** | | ChiCTR | | ChiCTR |
| **Weinreich-2021a, (phase 1-2)** | | NCT, EUCTR | | EUCTR |
| **Weinreich-2021b, (phase 1-2)** | | NCT, EUCTR | | EUCTR |
| **Weinreich-2021c, (phase 3)** | | NCT, EUCTR | | EUCTR |
| **Yu-2021b (PRINCIPLE)** | | ISRCTN, EUCTR | | ISRCTN, EUCTR |

Footnotes:

* Unpublished study, EA date reported on the trial registry record.

^a^ n = 74 published RCTs, several RCTs were registered more than once and/or in different registries.
