# Supplementary material for: Investigation of ethics approval as part of a research integrity assessment of randomised controlled trials in COVID-19 evidence syntheses: a meta-epidemiological study
Source: BMJ Open. 2025 Mar 24;15(3):e092244. doi: 10.1136/bmjopen-2024-092244 (PMC11934354; doi:10.1136/bmjopen-2024-092244)
Supplement: Supplementary file 5 [file bmjopen-2024-092244.reviewer_comments.pdf]

## PEER REVIEW HISTORY

BMJ Open publishes all reviews undertaken for accepted manuscripts. Reviewers are asked to complete a checklist review form and are provided with free text boxes to elaborate on their assessment. These free text comments are reproduced below.

### ARTICLE DETAILS

**Title (Provisional)**

Investigation of ethics approval as part of a research integrity assessment of randomized controlled trials in COVID-19 evidence syntheses: a meta-epidemiological study

**Authors**

Weber, Florencia; Pscheidl, Tamara; Sydenham, Emma; Meybohm, Patrick; Weibel, Stephanie

---

### VERSION 1 - REVIEW

---

| | |
|---|---|
| **Reviewer** | **1** |
| **Name** | **Giubilini, Alberto** |
| **Affiliation** | **University of Oxford** |
| **Date** | **06-Oct-2024** |
| **COI** | **None** |

I think the issue addressed - the reporting of ethics review in RCT for covid-19 and in systematic review. I should say my background is in philosophy and I work in applied ethics, and so I cannot really comment on the core aspects of this study, especially the methodology for the statistical analysis. So I am afraid all I can say is that the issue is very relevant, as are the result, but I am afraid this is not going to be very helpful or a very solid justification for my positive recommendation.

---

| | |
|---|---|
| **Reviewer** | **2** |
| **Name** | **Kolstoe, Simon** |
| **Affiliation** | **University of Portsmouth, School of Healthcare Professions** |
| **Date** | **07-Nov-2024** |
| **COI** | **None** |

This is an interesting manuscript that highlights a relatively well known problem, summarised by the authors in their conclusion as: "The lack of standardized procedures and guidelines for reporting information regarding ethics approval in the primary study record impedes the assessment of ethics, and therefore, leaves producers of evidence synthesis with a high number of RCTs, which are not eligible for evidence synthesis (i.e. RCTs categorized as ‘awaiting classification’)."

This is an important issue especially if it is inadvertently preventing studies that otherwise might have relevant information from being included in evidence synthesis work. Thus said there is an existential element to this problem as in most cases systematic reviewers, journal editors, and peer reviewers have little choice but to rely on fairly generic statements that an ethics review has been conducted. The lack of national registries to check that the ethics review has indeed been conducted is certainly a problem recognised both here and elsewhere. Furthermore even if the reporting requirements are met, there is little chance of determining the adequacy of any ethics review or indeed whether the ethics review even looked at the same project that is being reported. This is a well know problem especially to journal editors, highlighted again by this paper, but difficult to work out how to address.

I think this manuscript merits publication so as to further illustrate the problem, but a few improvements can be made:

1) The definition of research integrity in the background section (and subsequently repeated in the discussion) is vague, and the reference links through to the NIH definition of research misconduct. A bit more needs to be said about how research ethics relates to research integrity because as written I do not think the relationship is either clear or accurate e.g. see https://doi.org/10.1080/08989621.2023.2239712

2) The researchers looked for mention of "Ethics Committees" but this is a fairly UK/European term - did they also take into account the US (and others) names for the same function "Institutional Review Boards"?

3) In the methods, under the section "Recognition status of ethics committees" the authors state: "An ethics committee should have a national recognition status and there should be a national government department responsible for recording the national recognition status." This needs further explanation as it depends on both national legislation and the RCT topic/subject as some RCTs will fall out of scope for national committees so could, entirely acceptably, be reviewed by local committees.

4) In the methods, section 5. Written informed consent, it is not clear why having "written" consent is significant given that many studies now collect this electronically. This needs to be evaluated/discussed.

5) In the section "Date of ethics approval", it is of note that in some countries (e.g. UK) RECs do not "approve" studies as this is a decision that legally sits with study sponsors. Instead it

may be more accurate here to state "Date of ethics opinion" as more of a catch all across all jurisdictions.

6) Generally point - there are a couple placeholders in the text for missing references, these need to be addressed prior to publication, and the manuscript would benefit from furthe proof reading/sub-editing. But overall the language is fine.

7) The discussion is currently too long making it a bit unfocussed. The ambiguity between the definition of research integrity & ethics from the Background section is again here. The section could probably be reduced by a quarter to a third so as to aid focus.

---

**Reviewer** **3**

**Name** **Joffe, Steven**

**Affiliation** **University of Pennsylvania, Division of Medical Ethics, Professor of Pediatrics**

**Date** **25-Nov-2024**

**COI** **None**

---

NOTE: page numbers are to those listed on the bottom of each page.

1. General and most important comment: The manuscript conflates reporting of ethics with reporting of ethics APPROVAL. It is really about the latter, not the former. Ethics is much broader than ethics approval (and, conversely, mere ethics approval doesn't assure that the study was in fact ethical). Two examples of this conflation are pg. 3, line 43 "Compliance of clinical studies with ethical standards," which should rather be "Compliance of clinical studies with ethical approval standards, " and the first line of the discussion (should be "Reporting of ethics approval…"). However, this conflation is pervasive throughout the manuscript and should be corrected throughout.

2. Pg 8, line 9: occasionally, regulations may allow RCTs to be conducted without written informed consent (could be verbal consent, or consent might be waived, as in some cluster-randomized trials in the United States). How did the analysis deal with such cases?

3. The first paragraph of the discussion says that half are "not eligible for evidence synthesis due to uncertainties." However, given lack of standards related to reporting of ethics approval, this doesn't seem right. Should systematic reviewers really exclude half of these articles based on lack of reporting of information that isn't included in any consensus policy or standard?

4. Pg 20, line 35, "The reporting of ethics…has improved little over the years." It's important to acknowledge that this analysis, restricted to COVID-19 related RCTs (conducted in a very unusual situation and perhaps more internationally diverse than prior studies) might not be generalizable to all studies in the contemporary era and therefore that comparisons should

be made with caution. More broadly, I was surprised to see that generalizability of this study wasn't addressed as a limitation in the discussion.

5. Pg 21, line 6, "The fact that the large international multicenter studies behaved similarly in the reporting of ethics as the small single-centre studies could be another indication of the lack of awareness of the importance of full reporting." Could also be an indication of the lack of standards for reporting of ethics approval.

6. Pg 22, line 6, minor: "This finding differs amply…" Please rephrase. More broadly, although the writing is generally good, there are areas throughout the discussion that need carefully copy editing.

7. I'm unsure if the manuscript requires an abstract; none is provided. I defer to the editor.

---

## VERSION 1 - AUTHOR RESPONSE

Reviewer: 1

Dr. Alberto Giubilini, University of Oxford

Comments to the Author:

I think the issue addressed - the reporting of ethics review in RCT for covid-19 and in systematic review. I should say my background is in philosophy and I work in applied ethics, and so I cannot really comment on the core aspects of this study, especially the methodology for the statistical analysis. So I am afraid all I can say is that the issue is very relevant, as are the result, but I am afraid this is not going to be very helpful or a very solid justification for my positive recommendation.

*Response: Thank you for your honest assessment of your expertise and recognition of the relevance of our study and its results.*

---

Reviewer: 2

Dr. Simon Kolstoe, University of Portsmouth

Comments to the Author:

This is an interesting manuscript that highlights a relatively well known problem, summarised by the authors in their conclusion as: "The lack of standardized procedures and guidelines for reporting information regarding ethics approval in the primary study record impedes the assessment of ethics, and therefore, leaves producers of evidence synthesis with a high

number of RCTs, which are not eligible for evidence synthesis (i.e. RCTs categorized as ‘awaiting classification’)."

This is an important issue especially if it is inadvertently preventing studies that otherwise might have relevant information from being included in evidence synthesis work. Thus said there is an existential element to this problem as in most cases systematic reviewers, journal editors, and peer reviewers have little choice but to rely on fairly generic statements that an ethics review has been conducted. The lack of national registries to check that the ethics review has indeed been conducted is certainly a problem recognised both here and elsewhere. Furthermore even if the reporting requirements are met, there is little chance of determining the adequacy of any ethics review or indeed whether the ethics review even looked at the same project that is being reported. This is a well know problem especially to journal editors, highlighted again by this paper, but difficult to work out how to address.

I think this manuscript merits publication so as to further illustrate the problem, but a few improvements can be made:

1) The definition of research integrity in the background section (and subsequently repeated in the discussion) is vague, and the reference links through to the NIH definition of research misconduct. A bit more needs to be said about how research ethics relates to research integrity because as written I do not think the relationship is either clear or accurate e.g. see https://doi.org/10.1080/08989621.2023.2239712

*Response: Thank you for noticing the wrong link. The reference is now corrected: https://grants.nih.gov/policy-and-compliance/policy-topics/research-integrity*

*We agree with the NIH definition of Research Integrity:*

*“Failing to uphold research integrity undermines the public’s confidence and trust in the outcomes of NIH supported research.*

*Research integrity includes:*

- *the use of honest and verifiable methods in proposing, performing, and evaluating research*
- *reporting research results with particular attention to adherence to rules, regulations, and guidelines*
- *following commonly accepted professional codes or norms”*

*Thank you for referencing your article. It was very helpful for distinction between 'ethical compliance' of a clinical trial, which refers to adherence to research ethics as determined by*

*ethics committees, and the process of 'obtaining ethical approval' by study investigators or funders.*

*To clarify this for the reader, we included the following sentence in the Background section along with reference to your article "We distinguish between the 'ethical compliance' of a clinical trial, which refers to adherence to research ethics as determined by ethics committees, and the process of 'obtaining ethical approval' by study investigators or funders.[9]"*

2) The researchers looked for mention of "Ethics Committees" but this is a fairly UK/European term - did they also take into account the US (and others) names for the same function "Institutional Review Boards"?

*Response: Yes, we did. We searched for 'ethics committee' (EC), 'institutional review board' (IRB), or 'research ethics committee' (REC). We specified this in the methods section: "Originally, the third domain of the RIA on ethics approval included five items for the assessment of RCTs,[11] i.e. reporting of an ethics approval statement in the primary study report, name and location of the ethics committee (EC) (or alternatively named, 'institutional review board' (IRB), 'research ethics committee' (REC)), national recognition status of the EC according to country, ethics approval number (EN),…"*

3) In the methods, under the section "Recognition status of ethics committees" the authors state: "An ethics committee should have a national recognition status and there should be a national government department responsible for recording the national recognition status." This needs further explanation as it depends on both national legislation and the RCT topic/subject as some RCTs will fall out of scope for national committees so could, entirely acceptably, be reviewed by local committees.

*Response: Thank you for pointing out this need for explanation. The RIA tool is developed for and limited to interventional RCTs of an investigational medicinal product (IMP). All the included and investigated RCTs were interventional RCTs of an IMP in humans and therefore all need ethics approval (opinion) by an authorized institution such as a national recognized EC. What we mean by national recognition is the requirement of ECs or IRBs to submit paperwork to a national authority to certify their presence. This process is outlined for the USA in the article (PMID 32851230). Some countries such as India make the documentation publicly available (https://cdsco.gov.in/opencms/opencms/en/Clinical-Trial/Ethics-Committee). We are indeed looking to see if the trials adhered to the legislation, and for example for the trials done in Pakistan we identified that the clinical trial regulations met an*

*international standard but some of the trials were not done according to the terms of the regulations.*

*To make it clearer for the readers, we have improved the following sentence in the Background section: "This paper is part of a meta-epidemiological study which applies a novel and non-validated tool, designed for a research integrity assessment (RIA) of interventional RCTs of an investigational medicinal product (IMP) in the context of an evidence synthesis[12] to a pool of RCTs included in COVID-19 systematic reviews." And further on in the methods section, 3. Recognition status of ethics committees: "Interventional RCTs of an IMP in humans need ethics approval (i.e. ethics opinion) by an authorized institution such as an EC.[9 13] The ethics committee should have a national recognition status and there should be a national government department responsible for recording the committee's national recognition status."*

4) In the methods, section 5. Written informed consent, it is not clear why having "written" consent is significant given that many studies now collect this electronically. This needs to be evaluated/discussed.

*Response: We also accepted electronically signed IC. We specified this in the methods section, 5. Informed consent: "We assessed if the primary study report included a statement on whether the RCT has obtained written informed consent (including electronically signed informed consent forms) from all participants or their relatives prior to enrollment."*

5) In the section "Date of ethics approval", it is of note that in some countries (e.g. UK) RECs do not "approve" studies as this is a decision that legally sits with study sponsors. Instead it may be more accurate here to state "Date of ethics opinion" as more of a catch all across all jurisdictions.

*Response: Thank you for this comment. We included a general definition of "date of ethics approval" in the methods section and repeated it at the beginning of the respective para in the results section and in the corresponding Table 4: "In this study, we also extracted and assessed additional items on ethics approval, which were not included in the original RIA, i.e. the date of ethics approval (also defined as the date of ethics opinion) and study start, and the timing of ethics approval."*

6) Generally point - there are a couple placeholders in the text for missing references, these need to be addressed prior to publication, and the manuscript would benefit from further proof reading/sub-editing. But overall the language is fine.

*Response: Thank you for your critical review. The place holders are deleted as none of the planned publications are already published. We did careful proof-reading of the manuscript.*

7) The discussion is currently too long making it a bit unfocussed. The ambiguity between the definition of research integrity & ethics from the Background section is again here. The section could probably be reduced by a quarter to a third so as to aid focus.

*Response: We have revised and shortened the discussion section.*

---

Reviewer: 3

Dr. Steven Joffe, University of Pennsylvania

Comments to the Author:

NOTE: page numbers are to those listed on the bottom of each page.

1. General and most important comment: The manuscript conflates reporting of ethics with reporting of ethics APPROVAL. It is really about the latter, not the former. Ethics is much broader than ethics approval (and, conversely, mere ethics approval doesn't assure that the study was in fact ethical). Two examples of this conflation are pg. 3, line 43 "Compliance of clinical studies with ethical standards," which should rather be "Compliance of clinical studies with ethical approval standards, " and the first line of the discussion (should be "Reporting of ethics approval…"). However, this conflation is pervasive throughout the manuscript and should be corrected throughout.

*Response: Thank you for this important comment. It is true that we used the terminology "ethics (compliance)" and "ethics approval" incorrectly in some places. We have corrected the manuscript throughout, beginning with the title: "Investigation of ethics approval as part of a research integrity assessment of randomized controlled trials in COVID-19 evidence syntheses: a meta-epidemiological study".*

2. Pg 8, line 9: occasionally, regulations may allow RCTs to be conducted without written informed consent (could be verbal consent, or consent might be waived, as in some cluster-randomized trials in the United States). How did the analysis deal with such cases?

*Response: Thank you for this comment. We accepted verbal informed consent if this was approved by an EC. Indeed, there are two RCTs in our study pool which obtained written OR verbal informed consent (please see Excel file on OSF, linked to the manuscript). We added this aspect to our manuscript. We added to the Methods section, 5. Informed consent: "We also accepted verbal informed consent, if approved by an EC". And to the Results section: "Of 188 RCTs, 165 published RCTs and all six unpublished RCTs included a statement that IC was obtained (Table 1); 163 obtained IC in written form and two RCTs obtained verbal or written IC which was approved by an EC (Dequin-2020, Derde-2021)."*

3. The first paragraph of the discussion says that half are "not eligible for evidence synthesis due to uncertainties." However, given lack of standards related to reporting of ethics approval, this doesn't seem right. Should systematic reviewers really exclude half of these articles based on lack of reporting of information that isn't included in any consensus policy or standard?

*Response: This is a heated debate. We aim to support this discussion with data from the real world, i.e information on ethics approval available for systematic reviewers in published reports of RCTs. On the way to trustworthy evidence, we as systematic reviewers must proof studies for research integrity. We believe that obtaining ethics approval should be one important aspect in this regard.*

*While we agree that the reporting of ethics approval is not included in the CONSORT statement, it is included in the Declaration of Helsinki, Good Clinical Practice, and similar frameworks. Ethics committee approval is a prerequisite for registering a trial in a WHO-affiliated trial registry (https://www.who.int/clinical-trials-registry-platform), and the specific information required to be submitted is included in the guidance document for trial registries, the WHO International Standards for Clinical Trial Registries, item 21: "Three data components have to be collected concerning the ethics review : • status of approval by at least one named ethics review committee (not approved, approved, not available); • date of approval; • name and contact details of ethics committee(s) (contact details: address, phone, email)."*
https://www.who.int/publications/i/item/international-standards-for-clinical-trial-registers

*Our study showed that reporting of ethical approval in RCTs is poor, and, as under-reporting cannot be excluded, the assessment of ethics approval as part of the RIA tool is currently not reliable. This conclusion is already included in the manuscript. We also gave reasons and potential solutions for the reporting issue. The lack of standardized procedures and guidelines for reporting information regarding ethics approval in the primary study record impedes the assessment, and therefore, leaves producers of evidence synthesis with a high number of RCTs which are not eligible for evidence synthesis (i.e. RCTs categorized as 'awaiting classification'). Updated reporting guidelines may improve this issue. Furthermore, we determined that trial registries play an essential role as providers of information pertaining to ethics approval and as sentinels, ensuring that RCTs are carried out according to available ethics regulations.*

*In the last part of the discussion, we already suggested a solution for the handling of studies "awaiting classification" which differs from the original RIA tool (i.e. not to use these studies in evidence synthesis): "Therefore, at present, we suggest to assess ethics approval in RCTs during a research integrity assessment included in the evidence synthesis process, however, not to exclude studies assessed as 'awaiting classification' from the study pool and meta-analysis, but instead perform sensitivity analysis to investigate robustness of the results."*

4. Pg 20, line 35, "The reporting of ethics…has improved little over the years." It's important to acknowledge that this analysis, restricted to COVID-19 related RCTs (conducted in a very unusual situation and perhaps more internationally diverse than prior studies) might not be generalizable to all studies in the contemporary era and therefore that comparisons should be made with caution. More broadly, I was surprised to see that generalizability of this study wasn't addressed as a limitation in the discussion.

*Response: We really much appreciate your comment, but we disagree with this comment to a certain extent, and the comment also disagrees with the one below. We agree that COVID-19 studies were conducted in unique circumstances. However, these studies were all conducted in the contemporary era and we are comparing our findings with studies which were conducted in the 1990s and 2000s and observing little improvement over time. We describe a mechanism for improving the situation, which is, including EC/IRB approval details in the CONSORT statement. We believe that if the CONSORT statement included ethics approval as a reportable item, there would be improvement over time across all trial reporting, including in the event of trials addressing a new illness. Many of the trials in our dataset were conducted by the preeminent research organizations worldwide. For many of the trials, members of the writing committee are internationally recognized experts in clinical trials (i.e. Pan 2020) who*

*are perhaps not in the habit of reporting ethics approval in their studies because they have never been prompted to do so.*

5. Pg 21, line 6, "The fact that the large international multicenter studies behaved similarly in the reporting of ethics as the small single-centre studies could be another indication of the lack of awareness of the importance of full reporting." Could also be an indication of the lack of standards for reporting of ethics approval.

*Response: Correct. But we hope that a minimal standard for reporting of ethics approval for interventional RCTs of investigational medicinal products will be available in the updated CONSORT checklist as members of ethics committees and regulatory agencies will be important stakeholders.*

6. Pg 22, line 6, minor: "This finding differs amply…" Please rephrase. More broadly, although the writing is generally good, there are areas throughout the discussion that need carefully copy editing.

*Response: We deleted "amply".*

7. I'm unsure if the manuscript requires an abstract; none is provided. I defer to the editor.

*Response: Yes, of course. We apologize for this mistake. Unfortunately, the abstract was missing in the submitted version, but is now included in the revised version.*

---

**VERSION 2 - REVIEW**

---

| | |
|---|---|
| **Reviewer** | **2** |
| **Name** | **Kolstoe, Simon** |
| **Affiliation** | **University of Portsmouth, School of Healthcare Professions** |
| **Date** | **27-Feb-2025** |
| **COI** | |

---

Thank you for engaging with my first review of the manuscript. Just to clarify my acceptance of your manuscript is not related to citing my work, so if you would rather not do so that is absolutely fine, however I did think the paper I mentioned was relevant!
